# Diagnostic imaging of cervical intraepithelial neoplasia based on hematoxylin and eosin fluorescence

**DOI:** 10.1186/s13000-015-0343-8

**Published:** 2015-07-25

**Authors:** Mario R. Castellanos, Anita Szerszen, Stephen Gundry, Edyta C. Pirog, Mitchell Maiman, Sritha Rajupet, John Paul Gomez, Adi Davidov, Priya Ranjan Debata, Probal Banerjee, Jimmie E. Fata

**Affiliations:** Division of Medical Women’s Health, Staten Island University Hospital, 475 Seaview Ave, Staten Island, NY 10305 USA; Electrical Engineering Doctoral Program, City College of New York, The City University of New York, 160 Convent Avenue, New York, NY 10031 USA; Department of Pathology, Weill Cornell Medical College, 525 East 68th Street, New York, NY 10065 USA; Department of Obstetrics and Gynecology, Staten Island University Hospital, 475 Seaview Ave, Staten Island, NY 10305 USA; Division of Geriatrics, Department of Medicine, Staten Island University Hospital, 475 Seaview Ave, Staten Island, NY 10305 USA; Department of Chemistry, College of Staten Island, 2800 Victory Blvd., Staten Island, NY 10314 USA; Department of Biology, College of Staten Island, 2800 Victory Blvd., Staten Island, NY 10314 USA

**Keywords:** Diagnostic imaging, Cervical neoplasia, Hematoxylin and eosin, Fluorescence imaging

## Abstract

**Background:**

Pathological classification of cervical intraepithelial neoplasia (CIN) is problematic as it relies on subjective criteria. We developed an imaging method that uses spectroscopy to assess the fluorescent intensity of cervical biopsies derived directly from hematoxylin and eosin (H&E) stained tissues.

**Methods:**

Archived H&E slides were identified containing normal cervical tissue, CIN I, and CIN III cases, from a Community Hospital and an Academic Medical Center. Cases were obtained by consensus review of at least 2 senior pathologists. Images from H&E slides were captured first with bright field illumination and then with fluorescent illumination. We used a Zeiss Axio Observer Z1 microscope and an AxioVision 4.6.3-AP1 camera at excitation wavelength of 450–490 nm with emission captured at 515–565 nm. The 32-bit grayscale fluorescence images were used for image analysis.

**Results:**

We reviewed 108 slides: 46 normal, 33 CIN I and 29 CIN III. Fluorescent intensity increased progressively in normal epithelial tissue as cells matured and advanced from the basal to superficial regions of the epithelium. In CIN I cases this change was less prominent as compared to normal. In high grade CIN lesions, there was a slight or no increase in fluorescent intensity. All groups examined were statistically different.

**Conclusion:**

Presently, there are no markers to help in classification of CIN I-III lesions. Our imaging method may complement standard H&E pathological review and provide objective criteria to support the CIN diagnosis.

## Background

Cervical cancer is the second most common malignancy among women worldwide, with 80 % of cases occurring in low-income countries [1, 2]. In the United States, cervical cancer screening programs are effective in preventing cancer and reducing related mortality [3]. However, there are significant areas that need improvement such as the correct histopathological grade classification of cervical intraepithelial neoplasia (CIN) using light microscopy [4, 5]. Most CIN grade I (CIN I) lesions are transient viral infections and as per guidelines require surveillance [3, 6, 7]. Lesions diagnosed as CIN II and above usually necessitate treatment, especially in women 30 years of age and older [3, 8, 9]. The threshold to treat a patient is based on histopathological classification of CIN, yet criteria for grading are subjective and have high inter- and intra-observer variability [3, 8, 9]. The CIN diagnosis is based on the assessment of the immature parabasal cell expansion within the epithelial thickness. In CIN I, the parabasal cells are confined to the lower 1/3 of the epithelium. In CIN II lesions, the immature cells are located between 1/3 and 2/3 of the epithelial thickness, and in CIN III the abnormal growth expands to the upper 1/3. Using these criteria, the identification and grading of CIN is difficult [10, 11]. Poor accuracy arises from the fact that CIN diagnosis relies on using standard light microscopy to subjectively identify the localization of mitosis, and the extent of the upward growth of abnormal cells [3, 10]. Yet, non-cancerous epithelial changes: cervical atrophy, squamous metaplasia and cellular atypia associated with inflammation, may appear like CIN and make the diagnosis even more difficult [3]. In a large multicentered study that used expert pathologists to re-examine over 1000 cervical biopsies [12], the inter-observer agreement was low with a κ of 0.54 (95 % CI, 0.50-0.58). The greatest number of diagnostic disagreements in this study was between normal cervical tissue and CIN I, in which only 30 % of negative cases were agreed upon by study experts.

Incorrect classification of cervical lesions has a major impact on patient care. Patients may either be over-treated or risk having a high grade dysplasia missed. Furthermore, there are economic issues associated with an inaccurate diagnosis of CIN. In the U.S., the annual healthcare cost to screen and treat HPV-related cervical disease is about $6.5 billion [13]. Inaccurate classification of a patient’s risk to develop cervical cancer impacts post-colposcopy and biopsy surveillance protocols, which radically affects cost. Therefore, there is an urgent need to develop unique methods to improve the histopathological diagnosis and grading of CIN.

Fluorescence spectroscopy is a promising diagnostic technique to examine the inherent auto-fluorescence of tissue and its spectral characteristics, which allow the discrimination of normal tissue from pre-cancerous or cancerous lesions [14]. Endogenous fluorophores such as cytokeratins, NADP, aromatic amino acids, lipo-pigments, and various proteins, such as collagen and elastin, all fluoresce upon light excitation [14–18]. Cell and tissue auto-fluorescence provides valuable information about the microenvironment during physiological and/or pathological states and can identify intracellular alterations in metabolism [17]. For instance, cellular transformation alters the light emission of mitochondrial fluorophores and, therefore, changes in epithelial-stromal interactions can be detected by the evident decrease in stromal collagen fluorescence as normal tissue progresses to a pre-cancerous lesion [17].

Detection of cervical lesions in vivo has been facilitated by the use of optical imaging [19–24]. In 2006, the Food and Drug Administration (FDA) approved the first *in vivo* optical imaging device for diagnosis of high grade cervical dysplasia (CIN II and III) during colposcopy [21]. Tissue auto-fluorescence and reflectance properties can mark abnormal areas. Similarly, ex vivo examination of cervical biopsies using fluorescence spectroscopy can be done but has not been sufficiently evaluated despite promising data [25].

The goal of our study was to examine the fluorescence spectrum of generally used hematoxylin and eosin (H&E) stained cervical tissue, to assess its diagnostic potential. Eosin is a fluorescent red dye, which is a brominated derivative of fluorescein. Protein–eosin complexes increase proportionally to the concentration of the protein present [26–29]. Given these properties of eosin, we set out to determine whether a unique fluorescent signature can be derived from H&E-stained tissue, such that normal tissue can be distinguished from abnormal, and that CIN I can be differentiated from CIN III. Our findings suggest that fluorescence imaging of H&E stained cervical tissue is a new method that may provide objective criteria to aid in the diagnosis of cervical lesions.

## Methods

### Cervical tissue samples

Colposcopy Clinic medical records were reviewed from two medical centers, Staten Island University Hospital (SIUH) and Weill Cornell Medical College, to find women that had undergone a biopsy for an abnormal PAP smear. A total of 111 slides were obtained diagnosed as Normal, CIN I, or CIN III. In this study in which fluorescence imaging was being evaluated to examine lesions likely to progress and compare them to lesions likely to regress, CIN II cases were not selected for review. This was done because CIN II cases are a heterogeneous group of lesions that either behave like CIN I or CIN III study [28], therefore for this exploratory study the focus was to evaluate cases in which biologic behavior is better defined. Cases of CIN I and CIN III were classified according the original criteria developed by R. Richart [28]. Nuclear atypia was considered to be diagnostic for dysplasia and the grading system was based on the expansion of the immature dysplastic basal cells within the epithelial thickness. Cases with expansion confined to the lower 1/3 of the epithelial thickness were classified as CIN I and cases with expansion to the upper 1/3 epithelial thickness were classified as CIN III.

SIUH-archived H&E-stained slides were re-examined to select biopsies that had classic histopathologic characteristics for each category [3]. Any equivocal specimens were excluded. Cases were obtained by consensus of at least 2 pathologists. Only cases that had HPV DNA testing with Hybrid Capture II (HC2, Qiagen, Baltimore, USA) for both high and low risk HPV were considered, to improve the correct classification of CIN 1 lesions. The Normal group consisted of biopsies that had normal histology, in addition to a normal PAP smear and a negative HPV HCII test. Cases of squamous metaplasia, severe chronic cervicitis and/ or atrophy were excluded. For Weill Cornell Medical College specimens, the histologic diagnosis of CIN I or CIN III was confirmed by positive KI-67 immunostaining and HPV DNA by SPF10 PCR-LiPA25. Cases of normal cervical tissue were obtained from hysterectomies for leiomyomata. HPV negativity was confirmed with negative HC II test.

This work was performed under the IRB protocol #: SIUH08-043, which was reviewed and approved by the North Shore LIJ Staten Island University Hospital IRB (FWA #00002417; 500 Seaview Avenue, Staten Island NY 10305). The study was exempted from the HIPAA requirement for authorization and granted a waiver of consent as the pathological specimens used were fully de-identified.

### H&E staining

Cervical tissue specimens: The formalin-fixed cervical tissue specimens were collected from the repository of each institution; the samples were serially cut, and the sections (about 4 microns in size) were stained with Hematoxylin and Eosin (H&E) by standard procedure using an automated H&E staining machine.

### Image capture

**Fig. 1:**
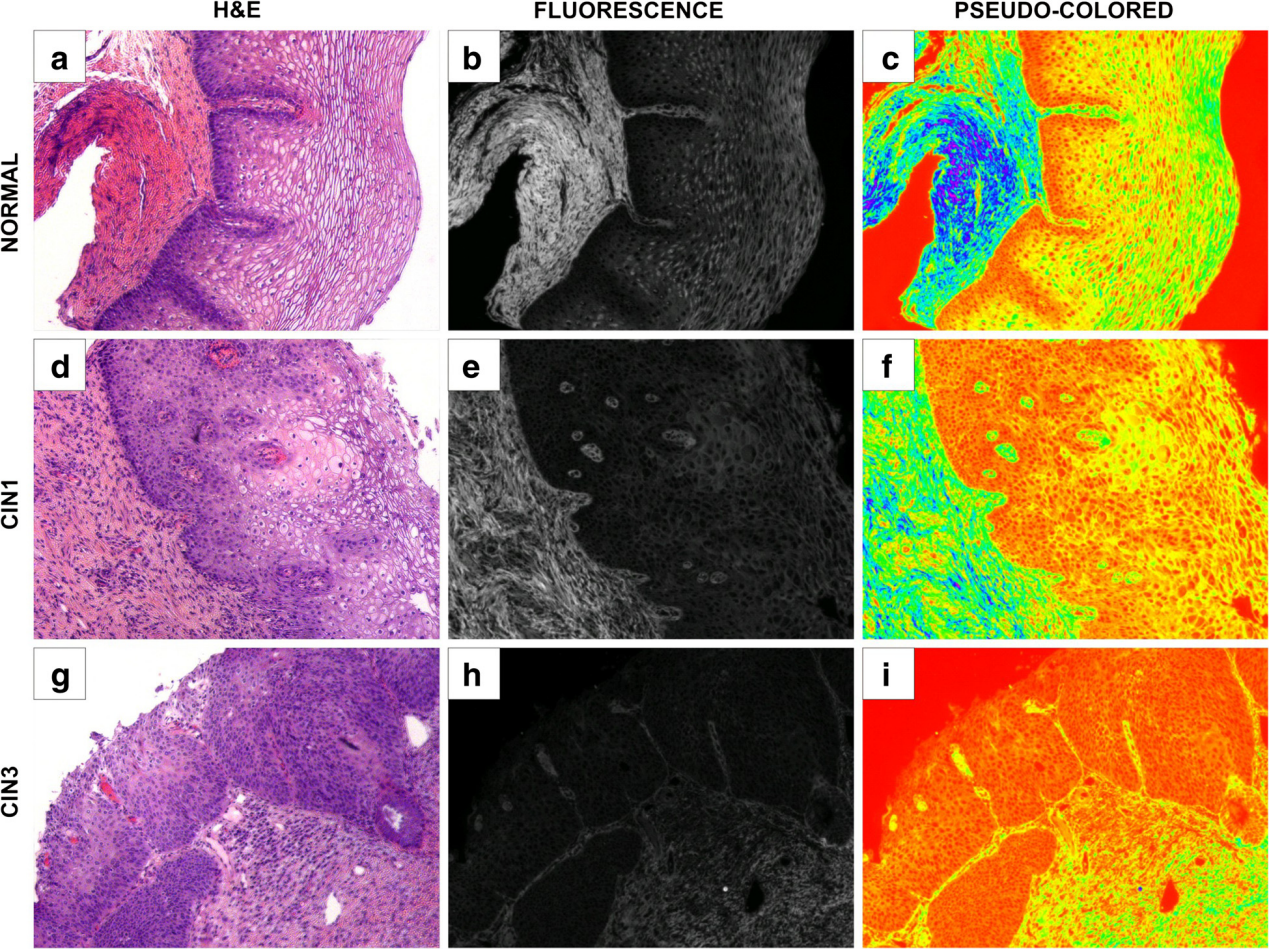
Fluorescent representation of normal, CIN I and CIN III cervical tissue specimens. **a**, **d** and **g** represent H&E stained cervical tissue. **b**, **e** and **h** represent inherent fluorescence of H&E sections at an excitation wavelength of 488 nm. **c**, **f** and **i** represent a color-map image in which fluorescent intensity is represented as shades (blue > green > yellow > red; high to low intensity). **a**, **b**, **c** = normal cervical tissue. **d**, **e**, **f** = CIN I cervical tissue. **g**, **h**, **i** = CIN III cervical tissue. Notice the lack of intensity in epithelium as you move from normal (**c**) to CIN I (**f**) to CIN III (**i**)

Images of the H&E stained cervical tissue sections were acquired using a Zeiss Axio Observer Z1 microscope and an AxioVision 4.6.3-AP1 camera using brightfield and at an excitation wavelength of 450–490 nm with emission captured at 515–565 nm (Fig. 1). The 32-bit grayscale fluorescent images were used for image analysis (see below). The same exposure time was used across all samples.

### Image analysis

**Fig. 2:**
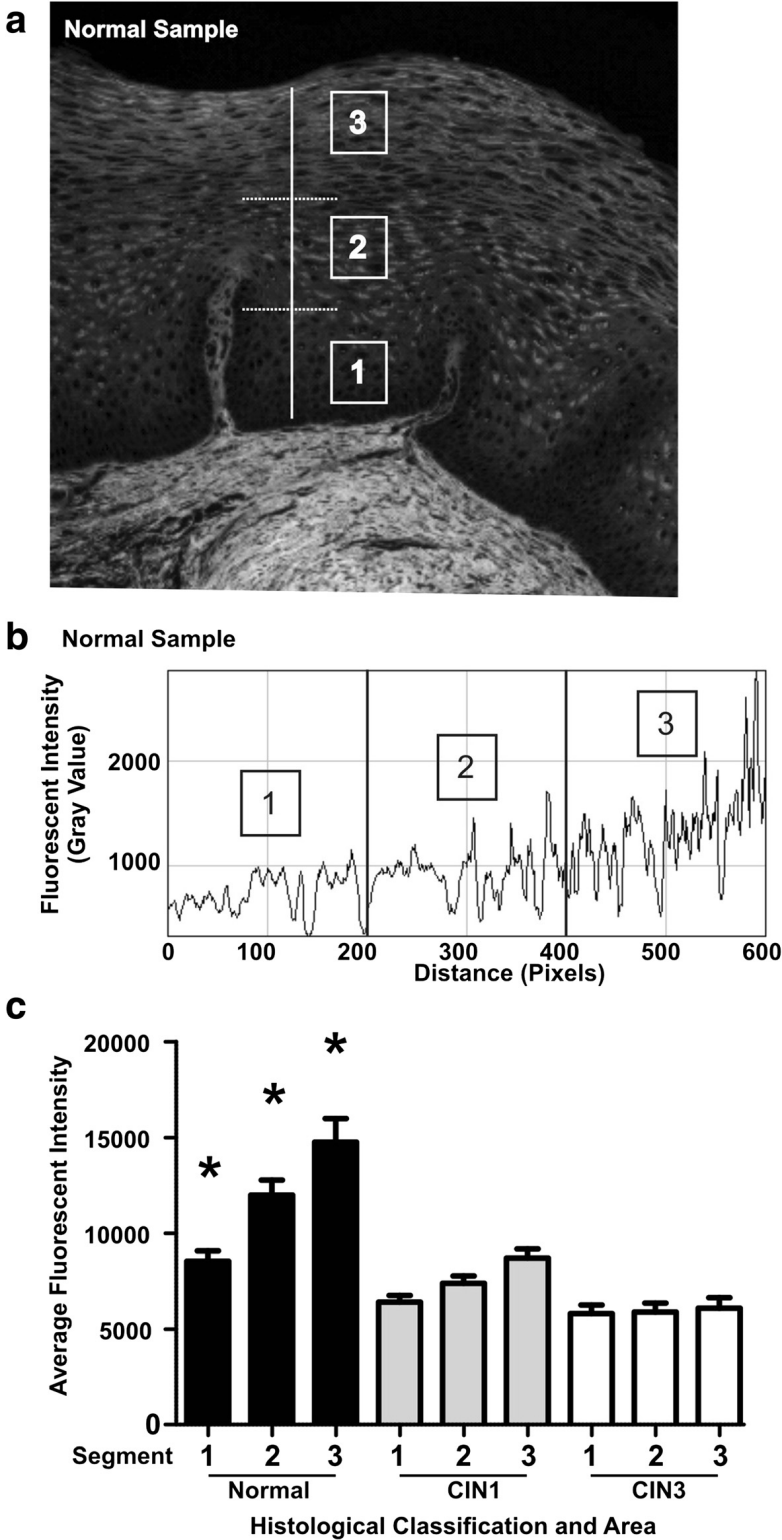
Quantifying average fluorescent values for normal, CIN I and CIN III cervical tissue. **a** Cervical epithelium divided into equidistant segments: segment 1 (bottom third), segment 2 (middle), and segment 3 (upper third). **b** Plotted fluorescent intensity profile of a line drawn from segment 1 to segment 3 on a normal sample. Segments are derived empirically by dividing the line distance into three parts. **c** Average fluorescent intensity for each segment derived from Normal, CIN I, and CIN III. Normal tissue is significantly higher in fluorescence intensity in all three segments when compared to CIN I and CIN III (p < 0.05). No significant differences were evident between CIN I and CIN III. Results are generated by averaging greater than 20 segmented lines for each sample. Total samples in each histological category are Normal = 13, CIN I = 18, CIN III = 12

32-bit grayscale fluorescent images were opened on ImageJ for image processing and analysis. ImageJ is a publically accessible image processing program developed at the National Institutes of Health. Firstly, multiple straight vertical parallel lines, originating from the epithelial basement membrane to the uppermost part of the surface epithelium, were drawn (Fig. 2a). The length of each line varied depending on the epithelium thickness (from basement membrane to surface epithelium). After each line was drawn, the ImageJ Plot Profile command was applied and the raw data were listed, copied and placed on a spreadsheet for post-analysis.

ImajeJ demonstrated the fluorescent intensity, using Gray Value, as it corresponds to the drawn line (Fig. 2a). Similar to how the epithelium is divided into areas using the CIN nomenclature (lower 1/3, middle 1/3, upper 1/3); the data from each drawn line was separated into 3 equidistant segments (Fig. 2a). For example, a line covering the full thickness of the epithelium having a length of 600 pixels would be divided into 0–200 (lower 1/3 segment), 201–400 (middle 1/3 segment), and 401–600 (upper 1/3 segment) (Fig. 2a). The data points from the drawn line as they correspond to the fluorescent intensity in each segment were plotted so that the X-axis represents the distance between the points on the drawn line measured by pixels and the Y-axis represents fluorescent intensity (Fig. 2b). The fluorescent intensity values were first analyzed without filtering any data points (Fig. 2b). Then, analysis was done by filtering lower values. The filtering removed variations caused by non-fluorescent black areas within the epithelial cells such as cytoplasmic glycogen (Fig. 2b). Due to cyclic estrogen effects, a threshold was derived in order to eliminate such cyclic variations (Fig. 2b).

The average fluorescence change throughout the whole epithelium was generated by obtaining the average fluorescence change in the lower, middle and upper segments. Three data points (one point representing each segment) were produced and used to plot a line that represents the overall average fluorescence change (Fig. 2c). It was found that the more fluorescent intensity exhibited by a tissue sample, the more the slope of the plotted line. Therefore, a tissue section that displayed increasing epithelial fluorescent intensities from basal to surface would have a higher slope (Fig. 2c higher line normal case) than a tissue sample that displayed no or minimal change in fluorescent intensity in that particular area (Fig. 2c middle and lower lines from CIN 1 & 3 cases). The slope of every line was normalized to remove sources that could cause sample variation, such as those caused by differences in H&E staining intensity or tissue thickness, images captured with different exposure times, and varying excitation energies. Normalization involved setting the average fluorescent intensity of the basal segment to a value of 1 such that all slopes originated from this value (Fig. 2c). This method provided an internal control for all tissue samples and eliminated potential sources of error.

### Statistical analysis

We analyzed data from each institution separately and jointly. To determine differences in fluorescent intensity between Normal, CIN I and CIN III, a One-Way ANOVA was performed followed by Tukey's Multiple Comparison Test. Student t-Tests were performed when comparing normalized slopes derived from the same histological type between each institution.

## Results

One hundred and eleven (111) formalin-fixed, paraffin-embedded, and H&E-stained cervical tissue specimens were used in this study; 47 Normal, 34 CIN I, and 30 CIN III cases (43 cases from SIUH, 68 cases from Weill Cornell Medical College).

Images of H&E-stained cervical biopsy specimens captured first with bright field illumination (Fig. 1a, d, g), then with fluorescent illumination (Fig. 1b, e, h) show distinct patterns of fluorescence. Pseudo-coloring of fluorescent images was achieved with Image J using the Spectrum Look-Up Table (Fig. 1c, f, i). Images were generated so that different fluorescent intensities were represented by colors. Using this color map, shades of blue correspond to the highest intensities and then decrease in order as follows: green, yellow, and red being the lowest.

**Fig. 3:**
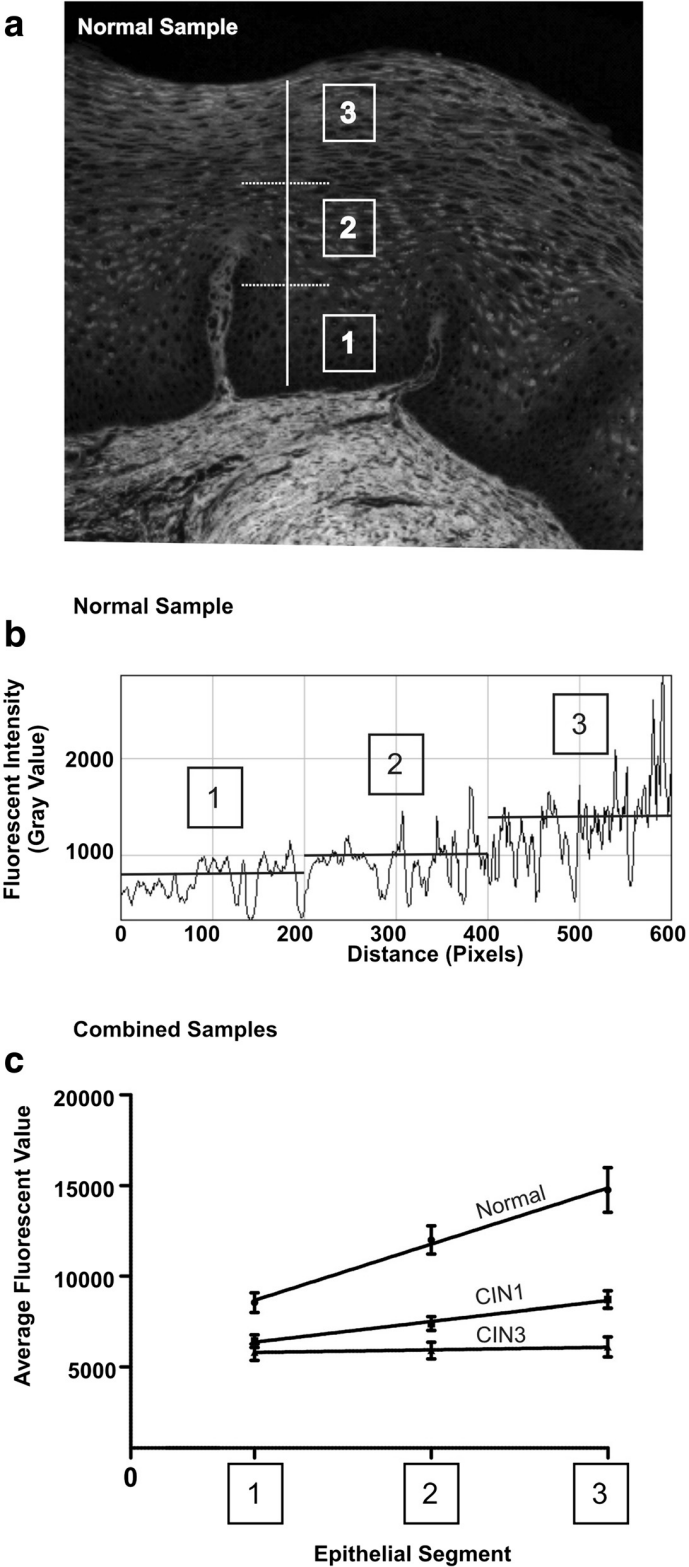
Linear regression analysis of segmental fluorescence intensity. **a** Cervical epithelium divided into equidistant segments: segment 1 (bottom third), segment 2 (middle), and segment 3 (upper third). **b** Fluorescence intensity profile of the line plotted in (**a**). Bisecting lines represent the 89th percentile of each segment. **c** The highest fluorescence intensity values (top 10 %) of each segment from (**b**) are averaged, plotted in (**c**) and analyzed with linear regression. Total samples in each histological category are Normal = 13, CIN I = 18, CIN III = 12

Pseudo-coloring of fluorescent images revealed considerable differences in the amount of epithelial fluorescence between Normal, CIN I, and CIN III (compare Fig. 1c with f and i). Normal tissue retained a significantly greater amount of epithelial fluorescence compared to the pathological tissues (CIN I and CIN III). The greatest intensity occurred in the cytoplasm of keratinocytes found in the superficial regions of the epithelium of normal cervix. Basal cells had the lowest intensity, while the fluorescence intensity increased progressively as keratinocytes matured and differentiated within the epithelium. CIN I exhibited higher epithelial fluorescence compared to CIN III (Fig. 1 compare f with i), but lacked the high intensity pattern seen in the superficial region of normal tissue. In CIN III, there was no significant increase in epithelial fluorescence, as the cytoplasmic fluorescence of all cells, from basal to superficial regions, was strongly diminished. To objectively quantify the epithelial fluorescent intensity seen on H&E tissue samples from SIUH, multiple straight lines (>20) were drawn from the lower segment to the upper segment using ImageJ line draw (Fig. 2a; normal tissue example). The ImageJ Plot Profile command was then applied to each line to obtain a fluorescent intensity profile (Fig. 2b); this data was stored for post analysis review. In the normal tissue samples provided, the fluorescent intensity plot was obtained from drawing multiple lines across the epithelium, as shown in Fig. 2a. The fluorescent intensity revealed a gradual increase in epithelial fluorescent intensity from segment 1 (lower) to segment 3 (upper). When reviewing the H&E slide and comparing it to the fluorescent image in Fig. 3a, epithelial cells in the latter had bright and dark areas. The dark areas primarily correspond to areas that are devoid of fluorescence, such as the nuclear region or cytoplasm containing glycogen. Many factors can affect the glycogen content of the cervical epithelium, such as menstrual cycle, therefore, to eliminate variation due to cyclical estrogen; a threshold was used to obtain the highest fluorescent values for each segment (lower, middle and upper; Fig. 3b). The analysis of fluorescence intensity for a particular line was done and compared in 2 % step-wise increments from the bottom up. When linear regression was applied to the plotted average fluorescent intensity line data across each segment, unique “signatures” for each histological type began to appear for the top 10 % and thus a threshold was derived to be at the 89^th^ percentile. By analyzing data in this manner, normal samples had a slope of 3110 ± 529 (fluorescent intensity value ± SEM), CIN I had a slope of 1144 ± 151, and CIN III had a slope of 145 ± 163. Statistical analysis (ANOVA followed by Turkeys’ pair-wise comparisons) on the slopes of each linear regression line revealed each was significantly different than the other (Fig. 3c; *p* < 0.05).

**Fig. 4:**
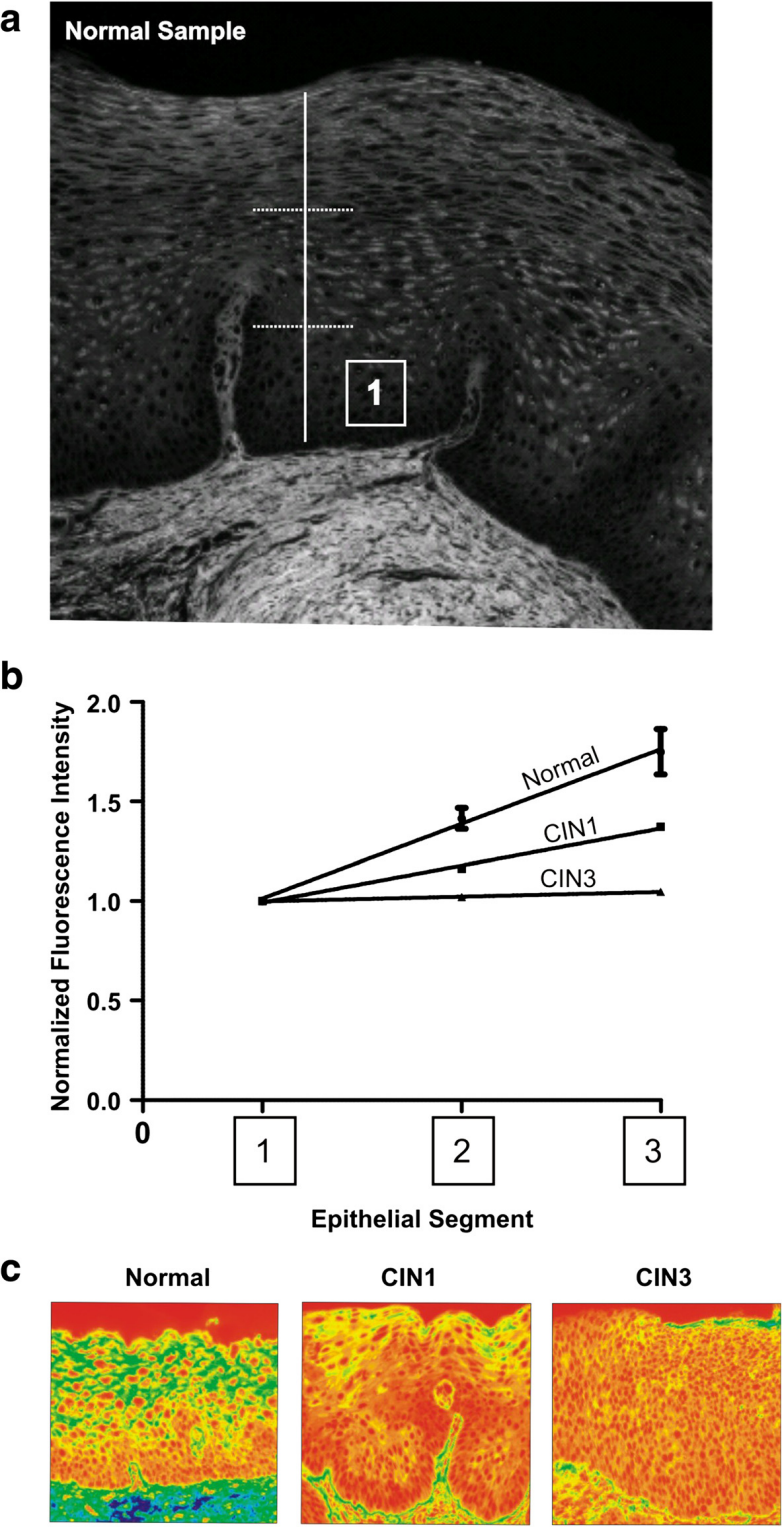
Normalizing linear regression to lower segment. **a** and **b** Fluorescent intensity averages in lower segments are set to 1 and all other data points are normalized to this value. Statistical analysis of the linear regression performed on normalized slopes indicates all histological types (N, CIN I, CIN III) are significantly different from each other (*p* < 0.05). Total samples in each histological category are Normal = 13, CIN I = 18, CIN III = 12. **c** Pseudo-colored intensity of fluorescent images from normal, CIN I, and CIN III epithelium that closely represent the average fluorescent intensity signature for each histological type

Normalization of slope data was developed to overcome the variation likely derived from a number of sources, such as the differences in H&E staining intensity, exposure time during image capture, fluorescent light sources, and thickness of tissue sample. Normalization involved setting all basal segment data points to 1 and adjusting all others on the same line relative to that change. This method provided an internal control for all tissue samples. This method therefore sets all slopes starting at 1 (normalize fluorescent intensity) regardless of potential sources of error (Fig. 4a, b). After normalization of slopes, significant differences between each histological type still remained (*p* < 0.05). Normal tissue had the highest normalized slope value (0.37 ± 0.06), followed by CIN I (0.19 ± 0.03), and then CIN III (0.02 ± 0.02). Pseudo-colored images from each histological type representing close approximation to these slopes are presented (Fig. 4c).

**Fig. 5:**
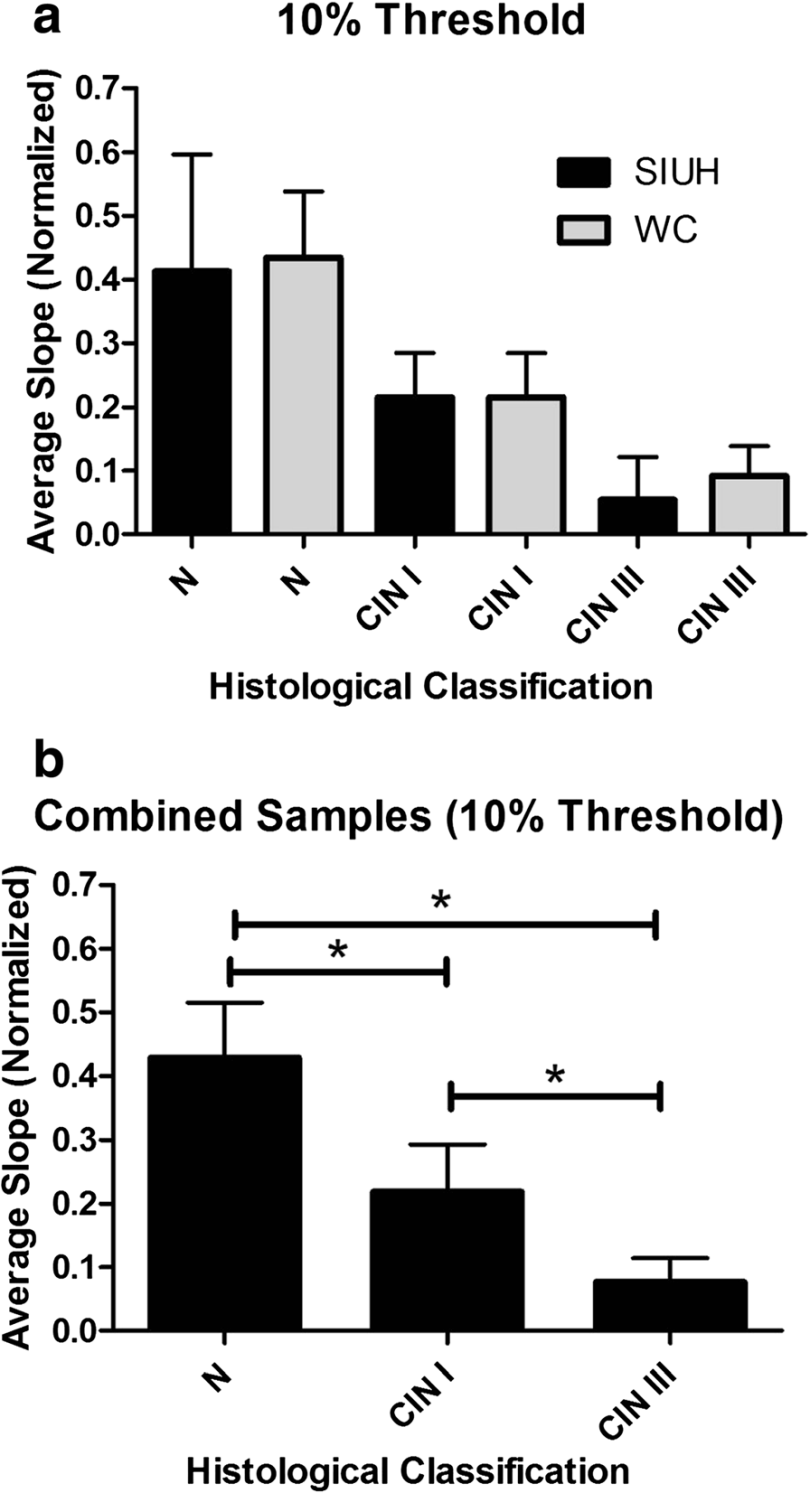
Validation of unique signatures. **a** An analysis of normal, CIN I and CIN III samples (N = 34, CIN III = 16, CIN III = 18) collected from a different institution (Weill Cornell Medical College) indicated that at a threshold of 10 % the slopes for normal and CIN I samples are not significantly different from the samples analyzed from SIUH. **b** When all samples from both institutions are combined, slopes from each histological type are significantly different from each other indicating a unique signature for normal, CIN I, and CIN III

Similar methods and results were obtained from Weill Cornell Medical College. In these samples, normal tissue had the highest slope (.41 ± .05), followed by CIN I (.22 ± .07), with CIN III (.10 ± .02) having the lowest slope. At the 89^th^ percentile threshold, no significant differences existed between samples analyzed from SIUH and Weill Cornell (Fig. 5a). More importantly, when all of the slides were combined between the two institutions (N = 47, CIN I = 34, and CIN III = 30), significant differences were evident between all histological classifications (Fig. 5b).

## Discussion

Fluorescence characteristics of H&E stained tissue specimens were described over 30 years ago [28]. Since then, only few investigators have used fluorescence spectroscopy to examine H&E-stained slides for the purpose of evaluating the tissue microenvironment. These studies have assessed the skin, pancreas, heart, spleen, colon, and kidney [27, 30, 28, 31–33]. They confirm the utility and reproducibility of fluorescence spectroscopy to characterize structures that are either difficult to visualize or not seen using standard light microcopy, yet become prominent with fluorescence imaging. Dinish et al. [34] examined H&E-stained pathology slides using fluorescence lifetime imaging microscopy and reported that tumor-associated molecules were retained in tissues despite fixation and staining. Fluorescence lifetime imaging properties were correlated with histological changes in tissue sections. Though fluorescence spectroscopy appears to be a promising method to evaluate the microenvironment of tissues, overall little work has been done in this area and no studies have examined if this method can aid in the diagnosis of pre-cancerous lesions of the cervix. Our findings indicate that unique fluorescent signatures exist between Normal, CIN I, and CIN III H&E-stained slides.

Our analysis and algorithm was first developed on cervical tissue specimens from Staten Island University Hospital (derivation set) and applied to a separate set of cervical specimens obtained from Weill Cornell Medical College (test set). The almost identical pattern between the derivation set and test set validates these unique fluorescent signatures (Fig. 5b). Our ability to normalize each slide with an internal control (segment 1) allowed us to remove any variations associated with slide preparation and staining, making possible comparisons among institutions. Essential to our study; was the use of well examined pathology cases determined to be ‘standards’, representing the categories of Normal, CIN I and CIN III cases. To overcome the difficulty in CIN diagnosis, we used specimens from two medical centers that obtained cases with two different approaches. The SIUH data set was obtained by consensus pathologic review in which selected cases had classic features for each histological category. This data set was previously reported and used for the evaluation of a marker of HPV-induced transformation [35]. In addition, the selected SIUH Normal cases had a negative HPV DNA test (Hybrid Capture II) for high and low risk viruses at the time of biopsy. The second set of slides, from the Cornell Medical College, was selected and reviewed by a gynecologic pathologist. The CIN diagnosis was confirmed by positive KI-67 immunostaining and PCR for HPV DNA. Both data sets represented a very different population of patients, yet the fluorescent signatures among histological categories were very similar when the threshold of 89^th^ percentile was used. The separation of the squamous epithelium into 3 segments was important for identifying a fluorescence pattern specific for each pathology group. It allowed us to evaluate the change in fluorescence. Normal tissue retained the greatest amount of epithelial fluorescence compared to the pathological tissues (CIN I and CIN III). In Normal group the basal and parabasal cells had low cytoplasmic fluorescence. However, fluorescence in the more mature and differentiated keratinocytes in the upper segments increased in intensity, probably due to the accumulation of various keratins and proteins associated with maturing keratinocytes [36]. Cells in superficial areas of normal epithelium (segment 3, Fig. 4b, c) acquired the highest amount of fluorescence intensity. This pattern was consistently visualized among all normal cases. This change in epithelial fluorescence was objectively quantified with the derivation of the slope data. In CIN III, a change in fluorescence intensity within the epithelium did not occur, as almost all of the upward expanding immature cells, from basal to superficial areas, had no or little enhancement of intensity. Therefore, CIN III cases had the lowest slope values (Fig. 4b, c). CIN I lesions demonstrated a pattern in which there was some increase in epithelial fluorescence but did not reach the intensity of normal cases (Fig. 4b, c). The high fluorescence of normal epithelial tissue is known to be in part due to keratin expression [15]. The lack of enhanced fluorescence within the epithelium of CIN III lesions is most likely attributed to the changes in the type of keratin expression associated with transformation of the cervical epithelium [37, 38]. Similarly, in CIN I the differential expression of HPV proteins among the different layers of the epithelium affects the maturation of keratinocytes [39] which may alter the pattern of fluorescence.

It is well known that in some situations normal cases are difficult to distinguish from CIN I, or III [4, 10]. The University of Virginia Health System conducted a study in which 1455 cervical biopsies were re-examined by expert consensus review and diagnoses compared to those by community pathologists. There was 86.5 % agreement on normal cases between experts and community pathologists, 61.9 % on CIN I lesions, and 75 % on CIN III. Biomarkers, p16^(INK4a),^ and Ki-67, were also evaluated to determine whether they could aide CIN diagnosis in community hospitals. Similar to other studies, p16^(INK4a)^ improved diagnosis of high grade CIN, but could not be used to distinguish CIN I from CIN III. Ki-67 also helped diagnosis of high grade CIN but less than p16^(INK4a)^ and the combination of Ki-67 and p16^(INK4a)^ was not better in this study than p16^(INK4a)^ alone [40]. Therefore, none of the markers currently available in the clinical setting can distinguish the critical cutoff of CIN I from CIN III. Yet, accurate histological grading of CIN is vital. In our study we found that with a 89^th^ percentile threshold, each histological category could be distinguished from another indicating a unique “signature” for Normal, CIN I, and CIN III. An appropriate diagnosis of CIN has significant impact on treatment and can affect clinical outcomes.

Seventy percent of CIN I lesions regress within one year [3], and are transient HPV infections, thus they require no intervention. In contrast, patients diagnosed with CIN III are likely to progress to cancer, therefore many of these patients are treated with loop electrosurgical excision procedure (LEEP), especially if they are 30 years or older [3]. In our study we were able to correctly classify normal, CIN I, and CIN III.

In the study from University of Virginia Health System [40], CIN II lesions had the lowest agreement, with only 47.6 % concordance between study and community pathologists. In this current exploratory study using fluorescence spectroscopy we wanted to examine lesions that are likely to progress from those that will regress, therefore we did not examine CIN II cases. This was done intentionally as experts believe CIN II behavior cannot be predicted by histology [28]. Now, that we have identified a unique fluorescent signature for CIN I and CIN III, our research is extended to focus on CIN II lesions. We are collecting cohorts of patients that were diagnosed with CIN II but were not treated in order to see if fluorescence imaging can predict outcomes.

Our present method has several advantages, the first of which is the ability to identify relevant areas of H&E-stained tissue sections that pathologists examine using conventional light microscopy. Fluorescence images are obtained from a standard H&E-stained slide without any other processing of the tissue and can be adopted by hospital laboratories without requiring extra-technical services. Inherent variations from the H&E staining are removed through our normalization process. Thus staining done at various times or at different institutions can still be compared and evaluated. Another advantage of fluorescence imaging using our method is that it overcomes the difficultly correlating abnormalities seen on H&E-stained tissue sections with subsequent special stained slides, where serial cut sections are required for staining. The original H&E and special stain slides represent the same region but not the same cells. Our method can directly image relevant areas on H&E slide to correlate fluorescence data with histopathology on light microscopy.

Fluorescent imaging of H&E slides may be a novel approach to evaluate cervical biopsies. In this test of concept study we examined an ideal data set, as each case represented classic lesion for each histology grade. The development of this method requires further systematic studies to determine its utility in resolving the current difficulties of CIN diagnosis. We are in the process of deriving standard fluorescent “signatures” for all CIN categories including mimics of CIN, like cervical atrophy, squamous metaplasia and cellular atypia associated with inflammation. Future studies will need to establish fluorescent signatures for these diagnoses as well.

## Conclusions

In conclusion, our diagnostic method uses standard H&E-stained tissue slides and provides quantitative information that could make the diagnosis of cervical dysplasia more accurate. We presented steps towards enabling quantitative pathology through the use of a normalized image-processing algorithm. The algorithm takes into account the concentration of eosin (via fluorescence imaging) as a function of spatial position (across the epithelium) and provides clinicians with a quantitative metric system that correlates with a diagnostic grade.
